# Surgical treatment of Denis type B thoracolumbar burst fracture with neurological deficiency by paraspinal approach

**DOI:** 10.1590/1414-431X20165599

**Published:** 2016-11-03

**Authors:** H. Wu, D.-X. Zhao, R. Jiang, X.-Y. Zhou

**Affiliations:** 1 Department of Orthopedics, China-Japan Union Hospital, Jilin University, Changchun, China; 2 Department of Operating Room, China-Japan Union Hospital, Jilin University, Changchun, China

**Keywords:** Thoracolumbar burst fracture, Paraspinal approach, Neurological deficiency, Surgery treatment

## Abstract

We aimed to describe the surgical technique and clinical outcomes of paraspinal-approach reduction and fixation (PARF) in a group of patients with Denis type B thoracolumbar burst fracture (TLBF) with neurological deficiencies. A total of 62 patients with Denis B TLBF with neurological deficiencies were included in this study between January 2009 and December 2011. Clinical evaluations including the Frankel scale, pain visual analog scale (VAS) and radiological assessment (CT scans for fragment reduction and X-ray for the Cobb angle, adjacent superior and inferior intervertebral disc height, and vertebral canal diameter) were performed preoperatively and at 3 days, 6 months, and 1 and 2 years postoperatively. All patients underwent successful PARF, and were followed-up for at least 2 years. Average surgical time, blood loss and incision length were recorded. The sagittal vertebral canal diameter was significantly enlarged. The canal stenosis index was also improved. Kyphosis was corrected and remained at 8.6±1.4^o^ (P>0.05) 1 year postoperatively. Adjacent disc heights remained constant. Average Frankel grades were significantly improved at the end of follow-up. All 62 patients were neurologically assessed. Pain scores decreased at 6 months postoperatively, compared to before surgery (P<0.05). PARF provided excellent reduction for traumatic segmental kyphosis, and resulted in significant spinal canal clearance, which restored and maintained the vertebral body height of patients with Denis B TLBF with neurological deficits.

## Introduction

Surgical procedures for thoracolumbar burst fractures (TLBFs) are performed through an anterior, posterior, or combined approach. These surgical approaches can be traumatic for patients (1,2). The treatment goals are the restoration of stability and alignment of the spine, but the optimal management for TLBF remains controversial (3,4). For a typical Denis type B fracture with neurological deficiency, decompression is considered necessary. This study was designed to describe a surgical technique that involves paraspinal-approach reduction and fixation (PARF) and to evaluate the outcome of TLBF managed with indirect reduction and posterior short-segment pedicle screw fixation without laminectomy and fusion in patients with Dennis type B fractures with neurologic deficits.

## Material and Methods

Between January 2009 and December 2011, a total of 62 patients were enrolled in this study, according to the following inclusion criteria: 1) single-level Denis type B TLBF confirmed with anteroposterior and lateral X-ray, computed tomography (CT), and magnetic resonance imaging (MRI); 2) neurologic deficits (Frankel A-D) independently confirmed with full neurological examination by at least two trained spinal surgeons at the time of admission; 3) age between 18 and 72 years at the time of injury; 4) admission to our hospital within 7 days after the injury.

Each patient's neurological status was evaluated through the Frankel scale. A visual analog scale (VAS) was used to assess back pain intensity. Radiographic assessments were performed using supine anteroposterior and lateral X-ray, CT, and MRI. These evaluations were performed at enrollment for all patients, and at 3 days, 6 months, and 1, 2 and 3 years postoperatively by a senior spinal surgeon. Incision length, operative time and blood loss parameters, as well as patient demographic and medical characteristics, were recorded after the selection process.

Vertebral kyphosis was measured from the superior endplate of the cephalic adjacent intact vertebra to the inferior endplate of the fractured vertebra. Disc height was defined as the mean of the anterior, middle and posterior heights of the disc on the lateral X-ray. Canal stenosis was determined using CT by directly measuring the anteroposterior canal dimension at the maximum area of the retropulsed osseous fragments. This value was compared with the average of similar dimensions measured at the levels above and below the injury level. The result of this comparison was reported as the anteroposterior canal stenosis index at the injury area.

This study protocol followed ethical standards and was approved by the institutional review board of our hospital. Informed written consent was obtained from each patient and their family.

## Results

**Figure 1:**
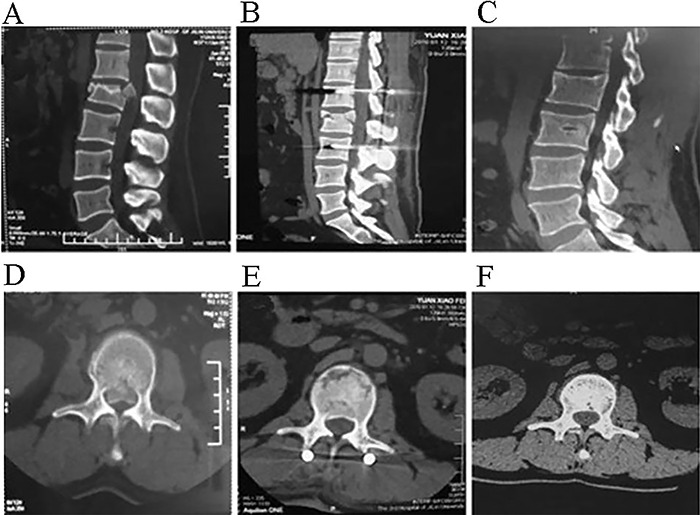
CT images showing significant restoration of the posterior and anterior vertebral height after surgery (*B*) and 2 years postoperatively (*C*), compared with the preoperative image (*A*). Postoperative axial CT image (*E*) showing significant canal decompression compared with the preoperative image (*D*). *F*, postoperative image two years after surgery showing that the canal was still enlarged.

All patients underwent successful PARF (male-to-female ratio: 3.86). The age of patients was between 18 and 72 years (mean: 42.3 years), and all completed the 2-year follow-up. The average follow-up duration was 28±7.4 months. All 62 patients had single-level fractures, comprising one at T11 level, 14 at T12 level, 30 at L1 level, 8 at L2 level, 6 at L3 level, and 3 at L4 level. The average surgical time, blood loss and incision length were 94.1±13.7 min, 91.6±16.9 mL, and 7.6±0.8 cm, respectively. Vertebral canal sagittal diameter was enlarged from an average of 5.7±1.6 to 15.2±1.2 mm (P<0.01). The canal stenosis index also improved from 41.0±1.3 to 97.8±0.6%. Kyphosis was corrected from 20.3±5.2 to 6.1±2.6^o^ (P<0.05), and remained at 8.6±1.4^o^ (P>0.05) 1 year later (Figure 1). Adjacent disc heights remained constant (Table 1). Average Frankel grades significantly improved at the end of follow-up. All 62 patients were neurologically assessed (grade A, n=3; grade B, n=0; grade C, n=1; grade D, n=6; grade E, n=52). The ten patients who were graded A-D had bowel or bladder disturbances. Three patients with a preoperative neurological status of grade A revealed no improvement at the latest follow-up, while all other patients had an improvement of at least one grade; 83.9% recovered to normal neurological status (Table 2). VAS pain scores decreased from 6.9±0.6 preoperatively to less than 1.5±0.8 (P<0.05) 6 months later. No serious complications were observed during follow-up.

**Table:**
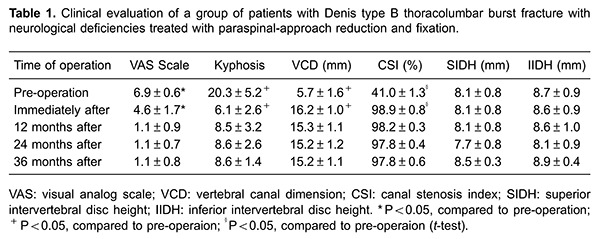

**Table:**
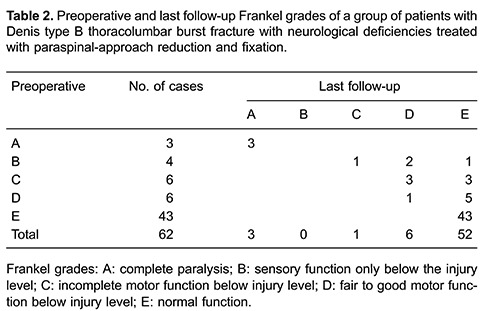

## Discussion

The selection of the surgical method for the treatment of TLBF remains a matter of debate (5,6). Multiple parameters such as the type and stability of the fracture, degree of canal compromise, injury to the posterior ligamentous complex and neurological status must be considered (7). Different surgeons choose different surgical approaches, which often depend on the surgeon's specific experience; and choices may not always be the most appropriate (6). How should the most appropriate approach be chosen? In our opinion, the following principles should be used: the approach should be based on the type of fracture, be familiar to the surgeon, and be minimally invasive. Every patient should be fully evaluated in order to make the best decision.

**Figure 2:**
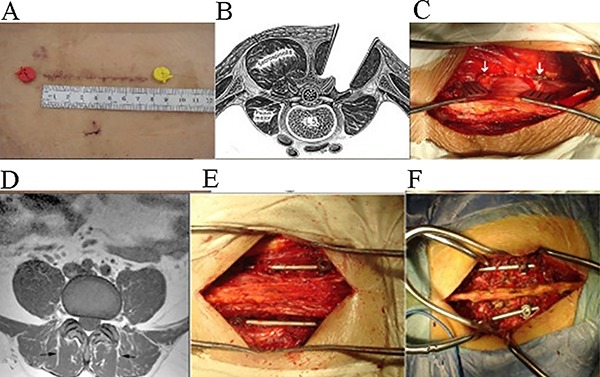
Surgical diagram. *A*, incision was minimized to 7-8 cm long. *B*, initial description by Wiltse of the paraspinal sacrospinalis-splitting approach to the lumbar spine showing the plane between the longissimus part and the multifidus part of the sacrospinalis muscle. *C*, facet joints are well exposed in the natural cleavage between the multifidus and the longissimus, which are the entering points of the pedicle screws. *D*, MRI shows the natural cleavage between the multifidus muscles. Paraspinal muscles are left intact in the paraspinal approach (*E*) compared with the traditional posterior approach (*F*). After surgery using pedicle and rod system internal fixation by the paraspinal approach, the composite of posterior column was preserved integrally (*E*).

The paraspinal approach was first used by Wiltse for lumbar spine fusion (8). We recently carried out a detailed study of this approach and expanded its application in the treatment of thoracolumbar fracture and other lumbar disorders (9). As shown in Figure 1, patients with TLBF and neurological deficits could achieve anatomic reduction through the paraspinal approach, which has the advantages of shorter incision length, less blood loss and shorter surgical time, compared with the traditional posterior approach. In all cases, there was a natural cleavage plane between the multifidus and longissimus muscles, which was the basis of the paraspinal approach. At T12 level, the muscle space was located approximately 1.5 cm from the midline, while at L4 level the space was approximately 3.0 cm from the midline. From this point, the transverse process and facet joint of T10-S1 could be easily exposed, and the pedicle screws could be precisely inserted (Figure 2B-D). This method of indirect reduction with short pedicle screw fixation without fusion provides another treatment option for TLBF with an intact posterior ligamentous complex. Other prospective studies have reported on pedicle screw fixation without fusion (10). Yang et al. (11) previously confirmed the immediate improvement in canal diameter achieved by indirect reduction with short-segment pedicle screw fixation without fusion within 2 weeks postoperatively. Paraspinal-approach instrumentation provides sufficient kyphosis reduction and reliable stability for the reconstruction of TLBF. In the present study, the vertebral canal diameter was significantly enlarged, and kyphosis was significantly improved. Conventional methods of repairing TLBF often involve laminectomy, which can result in further spinal instability (12). Results of the present study indicate that the paraspinal approach could be used in the treatment of most thoracolumbar fractures, of which even severe spinal canal occupation could be reduced, making this approach a good choice for Denis type B fractures. In addition, because the posterior longitudinal ligament is intact, decompression is not necessary, and anatomic reduction of the fracture can be obtained through appropriate vertical distraction (Figure 2) (13,14).

Cases such as those described in this study, can be treated by conventional decompression, reduction, fixation and fusion using the anterior, posterior, or both approaches. However, this may be an overly aggressive technique. Paraspinal-approach indirect reduction and fixation without fusion provides another treatment option for managing TLBF, in which there is an intact posterior longitudinal ligament and injury to the anterior and middle columns or to the anterior, middle and posterior columns (Denis type B). Determining an intact posterior longitudinal ligament is difficult, but can be achieved in two ways: directly from imaging studies, in which the fracture fragment of the vertebra near the canal does not flip; or by fluoroscopy after reduction with pedicle screws and rods, when the posterior edge of the fractured vertebra is parallel to the adjacent vertebral body. When attempting reduction *via* ligamentotaxis, pedicle screw insertion achieved with connecting rods should produce tension on the posterior longitudinal ligament and subsequent reduction of the fracture. If the fracture does not reduce with this technique, it may be because the posterior longitudinal ligament is damaged and does not provide the tension needed for reduction.

This study demonstrates the satisfactory clinical outcome of a series of neurologically impaired patients with selected Denis type B fractures treated with PARF. For patients with neurological deficiency, direct decompression has been routinely considered necessary before the reduction of the fracture. However, the present results demonstrate that the neurological status is not worsen with indirect reduction without decompression and fusion. Wilcox et al. (15) demonstrated that burst fractures are a dynamic event with maximum canal occlusion and maximum cord compression that occurs at the moment of impact, and that the fractures are poorly related to the final status, as shown on static images. Qiu et al. (16) used a finite element model of the T12-L1 motion segment to investigate the mechanism of burst fractures, and found that the canal encroachment at the end of the impact was less than the prior peaks. These findings explain the poor correlation between canal occlusion after trauma and neurological dysfunction. De Klerk et al. (17) reported a retrospective study, in which 42 patients with initial canal stenosis of >25% were managed conservatively, and followed-up by CT scans for 12-108 months after trauma. Obvious spontaneous remodeling occurred, and the degree of canal stenosis was reduced in all patients. An increasing number of studies have verified that there is no significant difference in neurological recovery between conservatively- and operatively-treated TLBF with canal compromise (18,19). It is not recommended to undertake surgical decompression for traumatic canal compromise in TLBF when there is a concern of static canal stenosis causing neurological dysfunction, or fear of neurological deterioration during rehabilitation. PARF can result in excellent reduction of TLBF in patients with neurological deficiency without decompression.

The paraspinal approach has many advantages compared with the traditional anterior and posterior approach: it results in less blood loss, shorter surgical duration, maintains the posterior ligamentous complex intact by preventing the stretching and distracting of paraspinal muscles, prevents denervation atrophy of the sacral spinal muscles by avoiding damage to the posterior branches of the lumbar nerve and dorsal branches of the lumbar artery, provides a broad operative field for the implantation of the pedicle screws, shorter bed rest time, and quicker recovery. In this study, fusion was not performed. After the surgery, the patient revealed a restoration of spinal motion, and thus, experienced a reduced risk of adjacent-level disease. Moreover, this method is less invasive and less complicated, compared with other conventional approaches. Furthermore, this approach is in accordance with the concept of a minimally invasive surgery, and can replace most posterior approach surgeries, which is worthy of further research and promotion.
